# Mitochondria Are Potential Targets for the Development of New Drugs Against Neutrophilic Inflammation in Severe Pneumonia Including COVID-19

**DOI:** 10.3389/fphar.2021.609508

**Published:** 2021-01-29

**Authors:** Nina V. Vorobjeva, Galina F. Sud'ina, Boris V. Chernyak

**Affiliations:** ^1^ Biology Faculty, M. V. Lomonosov Moscow State University, Moscow, Russia; ^2^ A. N. Belozersky Institute of Physico-Chemical Biology, M. V. Lomonosov Moscow State University, Moscow, Russia

**Keywords:** pneumonia, COVID-19, neutrophils, mitochondria, reactive oxygen species, NADPH oxidase, neutrophil extracellular traps, NETosis

## Introduction: Neutrophil Hyperactivation in Inflammatory Diseases

A high neutrophil-to-lymphocyte ratio is associated with disease severity and poor prognosis in pneumonia progressing to acute respiratory distress syndrome (ARDS) (Steinberg et al., 1994) including COVID-19 (Coronavirus Disease-19) caused by the novel SARS-CoV-2 coronavirus (Wang et al., 2020a; Mehta et al., 2020). Extensive infiltration of neutrophils into the pulmonary capillaries as well as their extravasation into the alveolar space have been described in bronchoalveolar lavage (Steinberg et al., 1994) and in autopsy specimens (Barnes et al., 2020; Wang et al., 2020b).

Neutrophils are the first line of defense against invading pathogens in the foci of inflammation, where they use effector functions such as phagocytosis, degranulation, and the formation of reactive oxygen species (ROS). Excessive activation of neutrophils results in the release of neutrophil extracellular traps (NETs) consisting of decondensed chromatin, «decorated» with myeloperoxidase (MPO), neutrophil elastase (NE), and other bactericidal proteins derived from intracellular granules (Brinkmann et al., 2004). The formation of NETs is usually accompanied by cell death, therefore, this process is called NETosis.

Examination of patients with severe pneumonia (Twaddell et al., 2019) as well as patients infected with SARS-CoV-2 (Zuo et al., 2020) revealed an increased level of NETosis markers, such as cell-free DNA, MPO-DNA-complexes, citrullinated histone H3, and a marker of cell death lactate dehydrogenase. In the serum from patients with COVID-19 the concentration of cell-free DNA correlated with the content of neutrophils, the marker of the acute phase of inflammation C-reactive protein, and the marker of thrombosis D-dimer (Zuo et al., 2020). This serum induced NETosis in healthy donor blood in an *in vitro* system (Barnes et al., 2020; Zuo et al., 2020). One of the manifestations of COVID-19 is Kawasaki syndrome, a vasculitis that occurs in children and is accompanied by excessive NETosis (Yoshida et al., 2020). NETosis in COVID-19 can be caused by epithelial and endothelial cells affected with the virus, by activated platelets, and by inflammatory cytokines. At the same time, excessive NETosis is involved in the development of the «cytokine storm» and immunothrombosis, which are the main cause of severe complications associated with COVID-19 (Wang et al., 2020a; Barnes et al., 2020), as well as with H1N1 influenza and some other viral infections (Cantan et al., 2019).

## The Role of Mitochondria in Neutrophil Activation

When activated, leukocytes accumulating in the lungs cause damage to the capillary endothelium and alveolar epithelium. Destruction of blood vessel endothelial cells can lead to blood coagulation and strokes (Ackermann et al., 2020). The attachment of neutrophils is mediated by adhesion molecules ICAM1, VCAM, etc., exposed on the surface of endothelial cells. The expression of these molecules is induced by inflammatory cytokines through the activation of the transcription factor NF-kB. We recently found that the expression of adhesion molecules is dependent on the production of mitochondrial ROS (mtROS), which contribute to NF-kB activation (Zinovkin et al., 2014; Galkin et al., 2016; Zakharova et al., 2017). Moreover, mtROS are critical for the increase in endothelium permeability and endothelial cell apoptosis induced by the inflammatory cytokine TNF (Galkin et al., 2016). The mitochondria-targeted antioxidants SkQ1 (10-(6′-methylplastoquinonyl) Decyltriphenylphosphonium) and SkQR1 (10-(6′-plastoquinonyl) decylrhodamine 19) protect endothelial cells *in vitro* and prevent increased expression of adhesion molecules and the lethal effect of TNF in a mouse model of systemic inflammatory syndrome (Zakharova et al., 2017). The mechanism of increased production of mtROS in the endothelium is not clear, but in the related model of endothelial stimulation with angiotensin II this mechanism has been shown to depend on mitochondrial permeability transition pore opening (mPTP) (Itani et al., 2016). Inhibitors of the mPTP are currently being developed and studied as potential drugs against cardiovascular diseases (Briston et al., 2019). It is possible that these drugs, when combined with mitochondria-targeted antioxidants, could be effective in preventing endothelial damage in a variety of inflammatory pathologies including pneumonia and COVID-19.

Our recent studies have shown that mtROS play a key role in neutrophil activation (Vorobjeva et al., 2017; Vorobjeva et al., 2020). The mitochondria-targeted antioxidant SkQ1 inhibited degranulation and ROS production induced by the chemoattractant fMLP via G-protein coupled receptor. It was concluded that mtROS are involved in the assembly and activation of the multicomponent enzyme complex NADPH oxidase (NOX2), which is the main source of ROS in neutrophils. The same cross-talk between mtROS and NADPH oxidase has been described in the endothelial cells (Nazarewicz et al., 2013). As in the endothelium, mtROS production in human neutrophils depends on the opening of mPTP (Vorobjeva et al., 2020). One of the important consequences of neutrophil activation is a delay in spontaneous apoptosis. This effect was blocked by mitochondria-targeted antioxidants (Vorobjeva et al., 2017), so it is possible that these agents could not only inhibit the damaging activity of neutrophils, but also reduce their number in inflammatory lesions.

Our experiments with the mitochondria-targeted antioxidant SkQ1 showed its potential efficacy against NETosis (Vorobjeva et al., 2020). It was shown that NETs formation induced by Ca^2+^ ionophore A23187 depends on the mPTP opening and the generation of mtROS. NETosis in this model was mediated by mtROS-dependent activation of NADPH oxidase. The massive production of ROS by NADPH oxidase, in turn, stimulated the opening of mPTP and mtROS generation. We have demonstrated that SkQ1 interrupted this vicious circle effectively neutralizing mtROS (Vorobjeva et al., 2017; Vorobjeva et al., 2020). The antioxidant MitoQ, which is structurally similar to SkQ1, suppressed NETosis in the mouse model of systemic lupus erythematosus (Fortner et al., 2020).

## Therapeutic Potential of Targeting Neutrophil Hyperactivation in Severe Pneumonia Including COVID-19

Various therapeutic approaches have been developed to inhibit neutrophils accumulation in the foci of inflammation, their activation and NETosis, as well as factors destroying NETs. They include anticytokine therapy directed against IL-1β (Anakinra; a recombinant IL-1β receptor antagonist is currently in clinical trials against COVID-19; https://clinicaltrials.gov: NCT04324021, NCT04330638, NCT02735707. 2020.), and NETosis may be one of its targets. Low molecular weight IL-8/CXCR2 antagonists have been tested in clinical trials for asthma, chronic obstructive pulmonary disease (COPD) and influenza, and have shown suppression of pulmonary neutrophilia and a decrease in the signs of NETosis (Narasaraju et al., 2020), but they have not been tested in COVID-19 trials. Among NETosis inhibitors, especially extensive research and testing has been conducted with NE inhibitors. The first of these, Sivelestat, has been approved for use against ARDS in Japan and South Korea, but meta-analysis of clinical data did not confirm its effectiveness (Tagami et al., 2014). New generation NE inhibitors have been clinically tested for the treatment of COPD and may hold promise for COVID-19, as well as recombinant DNAse I that degrades NETs (Narasaraju et al., 2020). The *in vitro* experiments have shown that NETosis can be prevented with the microtubule inhibitors. This group of drugs includes the oldest anti-inflammatory drug Colchicine, which, despite its strong cytotoxicity, is successfully used in small doses to treat acute gout and some other inflammatory diseases (Leung et al., 2015). Clinical trials of the efficacy of Colchicine against COVID-19 are currently underway (https://clinicaltrials.gov: NCT04326790, NCT04328480, NCT04322565, NCT04322682.2020.).

Leukotrienes, metabolites of arachidonic acid produced in the 5-lipoxygenase (5-LOX) pathway, are lipid mediators of inflammation involved in asthma and COPD, which are also considered potential targets for COVID-19 therapy (Funk and Ardakani, 2020). The 5-LOX pathway is activated in many diseases and triggers inflammatory responses that are not affected by glucocorticoids. Leukotriene B4, produced from leukotriene A4 by the soluble leukotriene A4 hydrolase, is a potent neutrophil chemoattractant that mediates the airway neutrophilia seen in severe COVID-19 (Wang et al., 2020b; Barnes et al., 2020). Another metabolite of arachidonic acid, epoxy fatty acids (EpFA), exhibits anti-inflammatory properties in contrast to leukotrienes. Prevention of NF-kB activation by EpFA can be mediated by inhibition of mPTP and subsequent production of mtROS (Wagner et al., 2020). To promote the accumulation of EpFA, inhibitors of soluble epoxide hydrolase (sEH), which converts EpFA to less active metabolites, have been developed (Wagner et al., 2020). An excellent effect in the resolution of inflammation was achieved by the double inhibition of leukotriene A4 hydrolase and sEH (Hefke et al., 2020; Hiesinger et al., 2020). Dual 5-LOX/sEH inhibition significantly suppressed leukocyte activation (Meirer et al., 2016), and neutrophil infiltration (Garscha et al., 2017). Another approach to the treatment of COVID-19, based on the use of the cysteinyl leukotriene receptor 1 (CysLT1) antagonist Montelukast, was recently proposed (https://clinicaltrials.gov/ct2/show/NCT04389411). In a small group of elderly patients with asthma, the use of Montelukast reduced the risk of SARS-CoV-2 infection (Bozek and Winterstein, 2020).

## Conclusion and Future Prospect

We hypothesize that mitochondria-targeted antioxidants and mPTP inhibitors may have beneficial effects in patients with severe COVID-19. These agents can be used alone or in the combination with other drugs. For example, the antioxidant N-acetylcysteine has recently been shown to enhance the action of Sivelestat against inflammatory pathology (Raevens et al., 2020). In summary, mitochondria appear to be a promising target for further drug development against severe pneumonia including COVID-19.

## Author Contributions

All authors listed have made a substantial, direct, and intellectual contribution to the work and approved it for publication.

## Conflict of Interest

The authors declare that the research was conducted in the absence of any commercial or financial relationships that could be construed as a potential conflict of interest.
